# 20 Hz temporal interference stimulation can more effectively enhance motor evoked potentials in the primary motor cortex

**DOI:** 10.3389/fnhum.2025.1524485

**Published:** 2025-02-19

**Authors:** Yajie Wang, Chunyue Zhu, Junhong Zhou, Tianli Fu, Jinlong Yan, Bangyu Wang, Jiaojiao Lü, Lingyan Huang, Yu Liu

**Affiliations:** ^1^Key Laboratory of Exercise and Health Sciences of the Ministry of Education, Shanghai University of Sport, Shanghai, China; ^2^Hebrew Senior Life Hinda and Arthur Marcus Institute for Aging Research, Harvard Medical School, Boston, MA, United States

**Keywords:** temporal interference stimulation, transcranial magnetic stimulation, primary motor cortex, frequency-dependent, motor evoked potentials

## Abstract

**Objective:**

This study investigates the effects of temporal interference stimulation (TI) with different envelope frequencies on the cortical excitability of the primary motor cortex (M1).

**Methods:**

In this randomized, double-blind, crossover study, 26 participants completed four separate study visits. During these visits, they received 20 min of three types of TI (10, 20, and 40 Hz envelope frequency) and sham stimulation applied over M1 in a randomized order. Transcranial magnetic stimulation (TMS) was employed to assess motor-evoked potentials (MEP) and resting motor threshold (RMT) over the left M1 (ipsilateral area) and right M1 (contralateral area) before, immediately after, and 30 and 60 min after stimulation.

**Results:**

The blinding efficacy was excellent, and no severe side effects were reported. TI stimulation with varying envelope frequencies affected MEP differently; 20 Hz TI stimulation enhanced the MEP of the ipsilateral M1 with after-effects appearing at 60 min, and no significant differences were observed between the 10 or 20 Hz TI stimulation with sham groups. However, no significant changes in RMT were observed under any of the TI conditions.

**Conclusion:**

20 Hz TI stimulation increased the cortical excitability of the ipsilateral M1, highlighting that frequency is an important factor in the modulatory effect of TI.

## Background

Temporal interference (TI) stimulation is an emerging noninvasive brain stimulation (NIBS) technique that modulates brain oscillations with higher spatial resolution and deeper penetration in subcortical regions than other NIBS techniques, such as transcranial alternating current stimulation (tACS) (Grossman et al., 2017; Rampersad et al., 2019; Zhu and Yin, 2023). TI is performed by attaching two pairs of stimulation electrodes to the scalp, delivering two high-frequency alternating currents with different frequencies through each pair. The superposition of these currents creates an electric field with a resulting modulation frequency (von Conta et al., 2022). Several studies (Ma et al., 2022; Zheng et al., 2024; Zhu et al., 2022) have reported the positive effects of TI on motor control and indicated that different stimulation parameters result in varying modulation effects (Gomez-Tames et al., 2021). For example, Zheng et al. (2024) found that repetitive 20 Hz TI on the human primary motor cortex (M1) increased vertical jump height in healthy adults, while Ma et al. (2022) showed that 70 Hz TI on M1 had no significant effect on motor learning in a serial reaction time task (SRTT). Thus, it is critical to determine the appropriate TI frequency to maximize functional performance benefits.

Motor control is dependent on numerous peripheral (e.g., muscles, and joints), spinal, and supraspinal elements, including cortical regulation within the brain. Studies have shown that the rhythmic oscillations in the cortical regions, including the MI, are important for motor control (Antal et al., 2016; Bologna et al., 2019; Feurra et al., 2011). Typically, increased power of alpha oscillations reflects the suppression of neuronal firing in regions that interfere with motor actions (e.g., 10 Hz) (Klink et al., 2020; Lange et al., 2014; Pollok et al., 2014; Vosskuhl et al., 2018). Additionally, changes in beta and gamma oscillatory activities are involved in various motor learning processes. An increase in oscillatory activity in the gamma band (e.g., 40 Hz) emerges in M1 during movement preparation and execution (Chen et al., 2022; Cheyne et al., 2008; Crone et al., 1998; Salenius et al., 1996). In contrast, oscillatory activity in the beta band (e.g., 20 Hz) increases during tonic contraction and decreases before movement onset and during movement execution (Engel and Fries, 2010; Gaetz et al., 2011; Jurkiewicz et al., 2006; Muthukumaraswamy, 2010). These neurophysiological characteristics of cortical oscillations at different frequencies, pertaining to different aspects of motor control suggest the importance of selecting the appropriate TI frequency. It is known that tACS influences the cortical excitability of M1 in a frequency-dependent manner. However, the effects of TI with different envelope frequencies on M1 have not been directly compared. This comparison provides direct evidence of the frequency-dependent effects of TI on motor control, informing the design for future studies.

Therefore, we explored the effects of TI with envelope frequencies of 10 Hz (alpha), 20 Hz (beta), and 40 Hz (gamma) on the excitability of M1 in a group of healthy adults. M1 excitability was assessed using motor evoked potentials (MEP) and the resting motor threshold (RMT) of transcranial magnetic stimulation (TMS). We hypothesized that compared to the sham group, TI stimulation with an envelope frequency of 20 Hz, which is the main oscillatory activity in human sensorimotor regions during rest, would induce a significant improvement in cortical excitability, as measured by MEP and RMT.

## Materials and methods

### Participants

Thirty healthy adult volunteers were recruited to participate in this study. The inclusion criteria were as follows: (1) right-handed, as determined by the Edinburgh Handedness Inventory (Oldfield, 1971) and (2) no history of psychiatric or neurological disorders (e.g., Parkinson’s disease and Alzheimer’s disease) or drug abuse. The exclusion criteria were as follows: (1) receiving NIBS within 1 month before the study; (2) any medication or psychotropic drugs used 4 weeks before the study; (3) the presence of mental implants or contraindications to electrical/magnetical stimulation (e.g., history of seizure); and (4) self-reported injuries related to motor control or history of lower limb injuries. The study protocol was approved by the Institutional Review Board of the Shanghai University of Sport, China (approval number: 102772021RT127). All participants provided written informed consent before participating in this study.

### Protocol

In this double-blinded, randomized, and crossover study, participants completed one screening visit and four study visits at the Biomechanics Laboratory of Shanghai University of Sport. During each study visit, they received 20 min of TI stimulation with envelope frequencies of 10, 20, 40 Hz, or sham stimulation in a randomized order. These study visits were separated by at least 3 days. Each visit was completed at approximately the same time of day. MEP and RMT were assessed before, immediately after (T0), 30 min after (T30), and 60 min after (T60) the stimulation.

### Assessment of MEP and RMT using TMS

**FIGURE 1:**
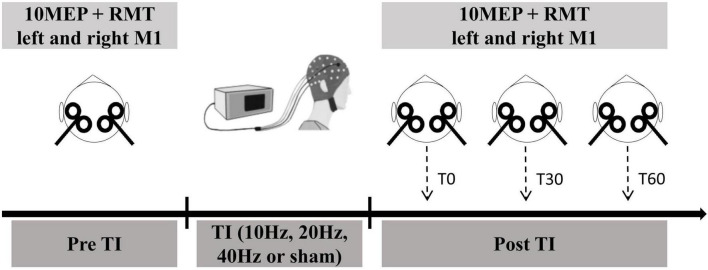
Study procedure. During each study visit, they received 20 min of TI stimulation or sham stimulation in a randomized order. TI stimulation was designated for one alternating current set at 2,000 Hz, while another current set at 2,010, 2,020, or 2,040 Hz (resulting in sinusoidal envelope frequencies of 10, 20, or 40 Hz). These study visits were separated by at least 3 days. 10 MEP and RMT were assessed before, immediately after (T0), 30 min after (T30), and 60 min after (T60) the stimulation.

The TMS with a figure-eight coil (loop diameter of 90 mm) connected to a monophasic Magstim Bistim^2^ system (Magstim 200, Whitland, Dyfed, UK) was employed to measure the MEP and RMT. The TMS coil was held tangentially over the scalp region corresponding to the left M1, with the coil handle pointing 45° posteriorly and laterally to the sagittal plane. MEP were recorded from the contralateral first dorsal interosseous muscle (FDI) using surface Ag/AgCl (42 mm × 25 mm) electrodes placed in a belly-tendon montage. A higher MEP suggests increased excitability and improved conduction along the corticospinal tract from the motor cortex to the muscles (Di Lazzaro et al., 1999). We determined the location of the FDI hotspot in the M1 as the spot that elicited the highest MEP with the lowest TMS intensity, as previously reported. The hotspot was marked to keep the coil position and orientation constant during subsequent measurement and interventions. The stimulation intensity was adjusted to evoke an MEP of approximately 1 mV peak-to-peak amplitude. Ten consecutive MEP at this intensity were used as a baseline, after which ten trials of the MEP at each time point were measured. The RMT of the FDI was defined as the lowest stimulus intensity that could elicit an MEP with an amplitude of 0.050 mV (peak-to-peak) in five out of ten pulses. Subsequently, MEP and RMT were verified in the contralateral stimulation area (right M1) using the same procedure. Each participant received a TI stimulation lasting 20 min. The same test procedure as the baseline test was performed immediately (T0), 30 min (T30) and 60 min (T60) after stimulation (Figure 1).

### Temporal interference stimulation

**FIGURE 2:**
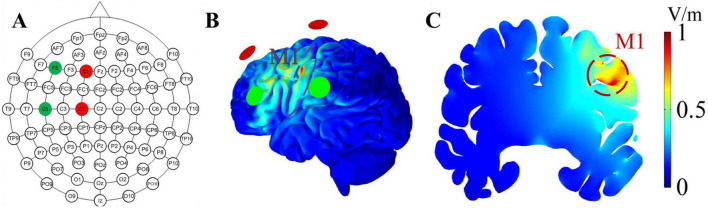
Electrode placement and the distribution of the envelope electric field. **(A)** The red and green circles represent the stimulation points for two alternating currents; red represents one channel of alternating current, while green represents the other channel. **(B,C)** illustrates the envelope electric field distribution on the cortical surface and coronal plane, respectively. The shades of red and blue denote different electric field intensities, with a stronger intensity being indicated by a shift toward the red color. The achieved envelope electric field amplitude in M1 at the intersection point is 0.78 V/m.

The design of the TI stimulation system followed a validated model (Zhang et al., 2022). It comprised multiple components, including a MATLAB program tailored for this study that generated digital signals for TI stimulation. Subsequently, these signals were output through a converter (USB-6361, National Instruments, Austin, TX) and delivered as electrical currents via an A395 linear stimulus isolator (A395, World Precision Instruments, Sarasota, FL, USA). The system can program and send alternating currents through four channels. The stimulation montage (e.g., current intensity and placement of electrodes) was determined by constructing the head model using the SimNIBS framework. Specifically, we segmented tissues and assigned conductivities, placed electrodes following the standard 10-10 EEG system of 64 channels, performed finite element meshing, and then calculated the electric field. In this study, the stimulation target was the M1. To achieve this, four identical round silicone electrodes (each measuring 1.5 cm in diameter) were set on C1, F1, C5, and F5 (Figure 2). Specifically, C1 and F1 were designated for one alternating current, while C5 and F5 were allocated for another current, with frequencies generated by channels C1 and F1 set at 2,000 Hz, and frequencies generated by C5 and F5 set at 2,010, 2,020, or 2,040 Hz (resulting in sinusoidal envelope frequencies of 10, 20, or 40 Hz). The electric field stimulation diagrams are shown in Figures 2B, C. Before the stimulation, reducing impedance through scalp abrasion with abrasive paste and applying conductive gel to ensure device resistance was under 15 kΩ. The peak-to-peak amplitude of the current was 2 mA for each pair of electrodes and the total stimulation duration was 20 min, including a 30-s ramp-up at the beginning and 30-s ramp-down at the end. However, the sham stimulation had only 30 s of current ramp-up and ramp-down at the beginning and end of the stimulation, respectively, and no current input during the intervening 19 min intervention (Yang et al., 2024).

During stimulation, participants sat in a comfortable chair in a quiet room, kept their eyes open, and were asked not to speak or move significantly. At the end of the visit, participants were required to complete questionnaires to assess the blinding efficacy and side effects.

### Statistical analysis

Statistical analyses were performed using SPSS Statistics 29.0 (IBM, Armonk, NY). The Shapiro–Wilk test was used to test the normal distribution of the outcomes. Normally distributed data were presented as mean ± standard deviations (SD). When the data met the assumptions of normality and homogeneity of variance, two-way repeated-measures analysis of variance (ANOVA) was used to examine the effects of TI stimulation on MEP and RMT. Model effects included stimulation conditions (10, 20, 40 Hz, and sham), time (baseline, T0, T30, and T60), and their interaction. The Greenhouse–Geisser correction was applied when Mauchly’s assumption of sphericity was violated. Post-hoc analysis with Bonferroni pairs was performed using significance models. The significance level was set at *p* < 0.05, and the effect size was estimated using partial eta-squared (*ηp*^2^).

Separate one-way ANOVA models were used to compare the percentage change [percent change = (post-stimulation—baseline)/baseline] for each outcome at each time point (e.g., T0, T30, and T60) between stimulation conditions.

Blinding efficacy was determined using the chi-square test. To examine the side effects induced by stimulation, Kruskal–Wallis tests were used to compare ranked categorical variables described as numbers: side effects (e.g., pain, itchiness, and burning) as reported by participants in the four groups based on information from the side effect questionnaire.

## Results

Twenty-six participants completed the study. Four participants withdrew before completing any of the stimulation visits, with three citing lack of interest and one due to technical difficulties with the equipment and were thus excluded from the analysis. Data from the remaining adults (13 males, mean age: 22.42 ± 2.12 years, mean height: 171.02 ± 9.14 cm, mean weight: 62.17 ± 9.74 kg) were included in the analysis. There were no significant between-group differences in MEP or RMT at baseline.

### The effects of TI stimulation on MEP

Two-way repeated-measures ANOVA models demonstrated significant interactions between stimulation and time (Table 1) [*F*_(9, 225)_ = 2.138, *p* = 0.027, *ηp*^2^ = 0.079], and a main effect of stimulation on the ipsilateral MEP [*F*_(2.2, 56)_ = 5.265, *p* = 0.006, *ηp*^2^ = 0.174]. *Post hoc* analysis revealed that in 20 Hz TI stimulation, the MEP was higher at T30 compared to baseline (1.06 ± 0.12 vs. 1.44 ± 0.67, *p* = 0.049) and in 10 Hz TI stimulation (1.07 ± 0.46 vs. 1.44 ± 0.67, *p* = 0.031). Additionally, at T60, the MEP was higher than in the sham condition (0.9 ± 0.43 vs. 1.36 ± 0.64, *p* = 0.015). No significant interaction between stimulation and time or main effects of stimulation and time on contralateral MEP were observed (*p* > 0.05).

**TABLE 1:** The effects of TI stimulation on MEP and RMT.

	Time condition				Group*time		
	Baseline	T0	T30	T60	*F*	*P*	η *p^2^*
**MEP (mV)_I**							
Sham	1.06 ± 0.12	1.01 ± 0.33	1.09 ± 0.41	0.90 ± 0.43	2.138	0.027^#^	0.079
10 Hz TI	1.04 ± 0.09	1.15 ± 0.45	1.07 ± 0.46^B^	1.18 ± 0.49^C^			
20 Hz TI	1.06 ± 0.12^A^	1.39 ± 0.78	1.44 ± 0.67^AB^	1.36 ± 0.64^C^			
40 Hz TI	1.04 ± 0.17	1.06 ± 0.38	1.21 ± 0.49	1.13 ± 0.52			
**RMT (% MSO)_I**							
Sham	31.62 ± 4.16	31.27 ± 3.88	31.62 ± 4.06	31.46 ± 4.18	0.563	0.827	0.022
10 Hz TI	31.38 ± 3.30	31.27 ± 3.38	31.42 ± 3.79	31.27 ± 3.45			
20 Hz TI	31.77 ± 3.77	31.69 ± 4.31	31.81 ± 3.96	31.58 ± 4.10			
40 Hz TI	31.58 ± 4.10	30.88 ± 3.73	31.15 ± 4.16	31.00 ± 4.08			
**MEP (mV)_C**							
Sham	1.08 ± 0.13	1.08 ± 0.46	0.96 ± 0.45	0.89 ± 0.37	1.251	0.265	0.048
10 Hz TI	1.06 ± 0.18	0.98 ± 0.41	1.11 ± 0.46	1.04 ± 0.51			
20 Hz TI	1.09 ± 0.15	0.87 ± 0.42	1.05 ± 0.48	1.00 ± 0.38			
40 Hz TI	1.03 ± 0.12	0.95 ± 0.41	0.94 ± 0.41	0.99 ± 0.47			
**RMT (% MSO)_C**							
Sham	35.19 ± 4.54	35.27 ± 4.98	35.08 ± 4.96	35.27 ± 4.98	1.607	0.114	0.060
10 Hz TI	34.85 ± 4.12	34.62 ± 4.83	34.35 ± 4.67	34.46 ± 4.36			
20 Hz TI	35.35 ± 3.99	34.88 ± 4.41	34.65 ± 4.77	35.15 ± 4.47			
40 Hz TI	34.77 ± 3.94	35.27 ± 4.23	34.73 ± 4.31	34.69 ± 4.15			

^#^Indicates a significant interaction effect (*p* < 0.05). Different superscript letters (A, B, and C) indicate mean that were significantly different from another. I, Ipsilateral; C, Contralateral; MSO, maximal stimulation output; T0, immediately after stimulation; T30, after stimulation for 30 min; T60, after stimulation for 60 min.

The percentage change in ipsilateral MEP after 20 Hz TI showed the largest change during each time condition (Table 2). Secondary one-way ANOVA models showed significant differences in the percentage change of ipsilateral MEP at T60 [*F*_(3, 103)_ = 3.077, *p* = 0.031]. *Post hoc* analysis revealed a significant difference between sham and 20 Hz stimulation (*p* = 0.022). No significant differences were observed at T0 or T30 (*p* > 0.05).

**TABLE 2:** The effect of TI stimulation on the percent change of MEP and RMT.

Time condition	10 Hz	20 Hz	40 Hz	Sham	F	*P*
MEP(T0)_I	0.12 ± 0.46	0.30 ± 0.73	0.04 ± 0.36	−0.04 ± 0.29	2.211	0.092
MEP(T30)_I	0.03 ± 0.45	0.32 ± 0.68	0.18 ± 0.47	0.04 ± 0.39	1.903	0.134
MEP(T60)_I	0.14 ± 0.50	0.28 ± 0.62^A^	0.11 ± 0.54	−0.15 ± 0.38^A^	3.077	0.031*
MEP(T0)_C	−0.04 ± 0.42	−0.19 ± 0.41	−0.05 ± 0.44	0.02 ± 0.46	1.085	0.359
MEP(T30)_C	0.09 ± 0.47	0.04 ± 0.57	−0.04 ± 0.47	−0.05 ± 0.50	0.456	0.714
MEP(T60)_C	0.07 ± 0.61	−0.04 ± 0.41	−0.04 ± 0.43	−0.09 ± 0.44	0.538	0.657
RMT(T0)_I	0 ± 0.05	0 ± 0.04	−0.02 ± 0.04	0.01 ± 0.03	0.694	0.558
RMT(T30)_I	0 ± 0.04	0 ± 0.04	−0.01 ± 0.04	0 ± 0.03	0.581	0.629
RMT(T60)_I	0 ± 0.06	−0.01 ± 0.05	−0.01 ± 0.05	−0.01 ± 0.03	0.313	0.816
RMT(T0)_C	−0.01 ± 0.04	−0.01 ± 0.04	0.01 ± 0.05	0 ± 0.04	1.204	0.312
RMT(T30)_C	−0.01 ± 0.04	−0.02 ± 0.05	0 ± 0.04	−0.01 ± 0.04	1.12	0.345
RMT(T60)_C	−0.01 ± 0.03	−0.01 ± 0.04	−0.01 ± 0.04	0 ± 0.04	0.324	0.808

^*^Significant difference. Different superscript letters indicate mean that were significant different from another. I, ipsilateral; C: contralateral; T0, immediately after stimulation; T30, after stimulation for 30 min; T60, after stimulation for 60 min.

### The effects of TI stimulation on the RMT

Two-way repeated-measures ANOVA models revealed no significant interaction between stimulation and time, nor any main effects of stimulation and time on the RMT of either ipsilateral M1 (*p* > 0.05), or contralateral M1 (*p* > 0.05, Table 1).

### Blinding efficacy and side effects

None of the participants reported serious adverse effects. Only mild-to-moderate side effects or uncomfortable feelings were reported (Table 3). The Kruskal–Wallis ANOVA models showed no significant differences in the number of participants who reported side effects among the four groups (all *p* = 0.410–0.824). The chi-square test showed that the blinding was successful (*p* = 0.489), with a total accuracy rate of 59.62%.

**TABLE 3:** Reported side effects in the four types of stimulation (%).

Side effects	10 Hz TI	20 Hz TI	40 Hz TI	Sham	*P*
**Pain**					
None	65.38% (17)	65.38% (17)	65.38% (17)	80.77% (21)	0.503
Mild	34.62% (9)	15.38% (4)	34.62% (9)	15.38% (4)	
Moderate	0	19.23% (5)	0	3.85% (1)	
**Itchiness**					
None	80.77% (21)	84.61% (22)	69.23% (18)	80.77% (21)	0.634
Mild	19.23% (5)	7.69% (2)	30.77% (8)	19.23% (5)	
Moderate	0	7.69% (2)	0	0	
**Burning**					
None	84.61% (22)	88.56% (23)	80.77% (21)	92.31% (24)	0.648
Mild	11.54% (3)	11.54% (3)	19.23% (5)	7.69% (2)	
Moderate	3.85% (1)	0	0	0	
**Skin redness**					
None	84.61% (22)	92.31% (24)	88.56% (23)	92.31% (24)	0.775
Mild	15.38% (4)	7.69% (2)	11.54% (3)	7.69% (2)	
Moderate	0	0	0	0	
**Sleepiness**					
None	50% (13)	34.62% (9)	46.15% (12)	38.46% (10)	0.768
Mild	23.08% (6)	34.62% (9)	30.77% (8)	46.15% (12)	
Moderate	26.92% (7)	30.77% (8)	23.08% (6)	15.38% (4)	
**Trouble concentrating**					
None	65.38% (17)	53.85% (14)	53.85% (14)	65.38% (17)	0.518
Mild	34.62% (9)	38.46% (10)	30.77% (8)	30.77% (8)	
Moderate	0	7.69% (2)	15.38% (4)	3.85% (1)	
**Acute mood change**					
None	73.08% (19)	80.77% (21)	73.08% (19)	80.77% (21)	0.824
Mild	23.08% (6)	15.38% (4)	23.08% (6)	19.23% (5)	
Moderate	3.85% (1)	3.85% (1)	3.85% (1)	0	
**Else**					
None	92.31% (24)	100% (26)	88.56% (23)	92.31% (24)	0.410
Mild	7.69% (2)	0	11.54% (3)	7.69% (2)	
Moderate	0	0	0	0	

## Discussion

In this pilot study, we compared the effects of TI stimulation with envelope frequencies of 10, 20, and 40 Hz on the MEP and RMT of both the ipsilateral and contralateral M1. These frequency-dependent characteristics of TI indicate the importance of selecting an appropriate frequency for TI stimulation in future studies to maximize the benefits for functional performance in humans. Consistent with previous findings (Zhang et al., 2022; Zheng et al., 2024), no serious side effects were observed during TI stimulation, indicating its safety and effectiveness.

Compared to the sham, only beta-frequency TI significantly increased MEP in the ipsilateral M1, with this increase observed at 60 min post stimulation. This may suggest a delayed effect of TI stimulation, and it is possible that MEP changes induced by beta-TI may persist for hours or even days. Although the exact mechanism of TI has yet to be fully understood, a recent animal study (Liu et al., 2023) showed that a 7-day 20 Hz TI stimulation could enhance basal synaptic transmission and long-term potentiation (LTP) plasticity. Another study (Qi et al., 2024) showed that 20 Hz TI stimulation increased calcium release during motor control tests and dendritic spine density. Increased dendritic spine density supports more efficient synaptic transmission and connectivity. Based on these mechanisms, beta-TI enhances the excitability of M1. However, alpha and gamma TI stimulation did not produce significant changes compared to the sham group; this is consistent with observations in previous studies (Ma et al., 2022; von Conta et al., 2022). For example, von Conta et al. (2022) reported no change in motor performance or EEG activity following alpha TI stimulation in young individuals. One possible reason is that MEP, measured by TMS over M1, mainly reflects changes in cortical excitability (facilitation and inhibition) related to motor pathways (Rossini and Rossi, 2007), while the entrainment of alpha oscillations originates mainly in postcentral regions and is more closely related to sensory processes (Tamura et al., 2005). Additionally, the non-significant MEP changes with gamma TI may be due to participants resting rather than performing actual movements. According to entrainment theory, the applied frequency would not be able to amplify such a different endogenous rhythm in the resting state. Conversely, some studies (Giustiniani et al., 2019; Ma et al., 2022; Nowak et al., 2017) showed that gamma TI or gamma tACS can improve behavioral performance, which was not significantly related to changes in MEP. For example, Nowak et al. (2017) reported a strong correlation between motor learning and changes in GABA inhibition rather than changes in MEP. This indicates that the changes induced by gamma TI may be related to other underlying neurophysiological pathways instead of MEP (Guerra et al., 2022; Guerra et al., 2019; Nowak et al., 2017), which needs to be explored in future studies with larger sample sizes and advanced neuroimaging techniques.

None of the three types of TI stimulation induced significant changes in RMT. This could be due to the intrinsic low-pass filtering property of the neural membrane, which prevents neurons from being activated by very high-frequency stimulation (e.g., ≥ 1 kHz) by increasing the motor threshold. Gomez-Tames et al. (2021) employed a multiscale mouse brain model and showed that low envelope frequencies (e.g., 5, 10, 15, 20, and 50 Hz) did not alter the motor threshold, which, however, increased when facing a high carrier frequency (e.g., 1 to 4 kHz). Therefore, the high carrier frequency of TI stimulation at 2 kHz may not alter the RMT.

No severe side effects were observed, and only mild-to-moderate side effects were reported following TI stimulation, consistent with previous TI experiments on human participants (Zhang et al., 2022; Zheng et al., 2024). The uncomfortable sensations induced by electrical stimulation are mainly attributed to the activity of cutaneous receptor activity in the somatosensory system (Fertonani et al., 2015). Focused electrical stimulation activates fewer cutaneous receptors, thereby reducing sensations of discomfort (Sheffield et al., 2022). TI was more centralized than tACS, with less co-stimulation of cortical regions near the electrodes (von Conta et al., 2022). Notably, in the present study, TI was generated using a low frequency (2 kHz) carrier frequency, consistent with its initial application in rodents (Grossman et al., 2017), and further refinement for human applications (Zheng et al., 2024) to target both superficial and deep brain regions. Acerbo et al. (2024) conducted a study comparing intracerebral recordings obtained via sEEG between low- and high-carrier-frequency TI, reporting that the effect of TI was independent of the carrier frequency. Therefore, although it is highly likely (Vassiliadis et al., 2024), we cannot be entirely certain that safety, tolerability and blinding efficiency would be identical when using other higher carrier frequency. Future research could focus on evaluating the safety and effect elicited by TI across a broader range of parameters, including various carrier frequencies and intensities.

Future studies with larger samples are needed to confirm the observations of this study, and the time points for TMS measurements after stimulation should be extended. Additionally, research should explore appropriate parameters for TI protocols in other populations (e.g., older adults, athletes, and individuals with neurological conditions). In this study, only the effects of TI on MEP and RMT were assessed. Future studies employing behavioral outcomes (e.g., reaction times, movement accuracy) or neuroimaging (e.g., fMRI) techniques are needed to provide more detailed insights into the neural mechanisms underlying the frequency-dependent effects of TI stimulation. Finally, the sham stimulation had no current, thus, further studies are needed to add an “active sham” (Acerbo et al., 2024) group to verify whether the observed effect is due to the envelope frequency or the high carrier frequencies.

This study shows that beta TI over the left M1 improved cortical excitability, which was assessed by the MEP of the ipsilateral M1, whereas alpha and gamma TI did not alter cortical excitability. These findings highlight the importance of selecting the appropriate envelope frequency for modulating effects, potentially contributing to future therapeutic applications of TI.

## Data Availability

The raw data supporting the conclusions of this article will be made available by the authors, without undue reservation.
